# Genome Sequences of a Novel Picorna-Like Virus from Pacific Abalone (*Haliotis discus hannai*) in South Korea

**DOI:** 10.1128/genomeA.00484-17

**Published:** 2017-09-07

**Authors:** Jong-Oh Kim, Jong Kyu Lee, Yong Bae Seo, Han-Kyu Lim, Gun-Do Kim

**Affiliations:** a Institute of Marine Biotechnology, Pukyong National University, Busan, Republic of Korea; b Department of Marine and Fisheries Resources, College of Natural Sciences, Mokpo National University, Muan, Jeonnam, Republic of Korea; c Department of Microbiology, College of Natural Sciences, Pukyong National University, Busan, Republic of Korea

## Abstract

We report the draft genome sequences of a novel member of the *Picornavirales* isolated from Pacific abalone (*Haliotis discus hannai*). The full length of the assembled draft genome sequences, obtained by use of a next-generation sequencing technique, were 8,019 nucleotides, including an RNA-dependent RNA polymerase gene (5,088 nucleotides) and a capsid protein gene (2,553 nucleotides). This genome sequence will be useful for understanding viral disease of Pacific abalone.

## GENOME ANNOUNCEMENT

Abalone is one of the commercially important marine gastropods (1). However, the aquaculture production rate of abalone has been decreasing recently, possibly due to several reasons, such as deterioration of aquaculture environments, genetic degradation, and diseases (2). A variety of marine viruses cause significant economic losses in an aquaculture species (3, 4). Until now, only abalone herpesviruses have been confirmed as the causative agents of the disease observed in farmed abalone (5). Therefore, understanding of host-virus interactions is an essential step for enhancing production for aquaculture. However, difficulties in viral identification have hindered research in this field, especially the demonstration of a fundamental relationship between the presence of a virus and the onset of the disease (6). In recent years, next-generation sequencing (NGS) technologies have profoundly improved the process of virus identification (7).

Soft tissue isolated from the Pacific abalone *Haliotis discus hannai* was homogenized in 1 mL of RiboEx (GeneAll, Daejeon, South Korea) in a glass homogenizer vessel, and RNA was isolated with chloroform and isopropanol (8). RNA was purified using a Hybrid-R column (GeneAll) according to the protocol provided by the manufacturer. Next-generation sequencing was carried out by Illumina HiSeq 2000 (Illumina, San Diego, CA, USA) according to the manufacturer’s protocol. The obtained raw read sequences were cleaned and assembled by the CLC Assembly Cell Package (version 4.2.0; CLCBio, Arhaus, Denmark) and CLC mapper (CLC Assembly Cell).

From the *Haliotis discus hannai* an 8,019-nucleotide (nt) contig with two continuous open reading frames was assembled, corresponding to the picorna-like virus. The picorna-like virus is a rapidly increasing taxonomic unit of positive-stranded RNA viruses that have wide-ranging hosts (9). The draft genome contains two open reading frames, RNA-dependent RNA polymerase (RdRp), which is located between 107 and 5,194 nucleotides (5,087 bp) from the 5′ end of the genomic RNA, and coat protein located between 5,402 and 7,954 nucleotides (2,552 bp) from the 5′ end of the genomic RNA. RdRp is composed of 1,695 amino acids, with a calculated molecular mass of 194.4 kDa. The translated open reading frame of RdRp aligned 28% by amino acids to Beihai picorna-like virus 64 (NCBI reference no. YP_009333388). The coat protein is composed of 850 amino acids, with a calculated molecular mass of 95 kDa. The translated open reading frame of coat protein aligned 37% by amino acids to Wenzhou picorna-like virus 24 (YP_009337258).

Further studies for taxonomic and functional analysis are required to completely clarify this virus. The genome information presented here may be useful to increase our understanding of viral disease in abalone aquaculture.

### Accession number(s).

The draft genome sequences of the picornavirus-like virus from a Pacific abalone (*Haliotis discus hannai*) have been deposited in GenBank under the accession no. KY933456.
